# From community diagnosis to policy dialogue: co-creating childhood obesity prevention strategies in rural and urban Portugal

**DOI:** 10.3389/fpubh.2026.1934381

**Published:** 2026-10-01

**Authors:** Tania Gaspar, Maria Rosa Nóbrega, Fábio Botelho Guedes, Ana Cerqueira, Leonor Quelhas Pinto, Miguel Telo de Arriaga

**Affiliations:** 1 HEI-Lab - Digital Human-Environment Interaction Labs, SPIC - Psychology, Innovation and Knowledge Service, Lusófona University, Lisbon, Portugal; 2 Aventura Social Association (ASA), Lisbon, Portugal; 3 Directorate-General of Health of Portugal (Direção-Geral da Saúde), Lisbon, Portugal

**Keywords:** childhood obesity prevention, co-creation, community engagement, participatory approaches, school-based health promotion, urban-rural disparities

## Abstract

**Introduction:**

Promoting healthy lifestyles from early childhood is central to balanced development and the prevention of health problems throughout life. The European HEALTH4EUKids project examines a participatory process of community diagnosis, co-creation into local policies for child health promotion and childhood obesity prevention in two Portuguese territories with distinct rural and urban profiles.

**Methods:**

A cross-sectional observational study was conducted within a mixed-methods action-research framework. The study provides an exploratory assessment of children's wellbeing indicators and contextual differences, without permitting causal inference. Participants included 202 primary-school pupils (mean age = 7.43 ± 1.14 years) and 54 community adults (mean age = 48.76 ± 11.51 years), alongside stakeholders from health, community, and local decision-making sectors. Data included a questionnaire adapted from the 2022 Health Behavior in School-aged Children (HBSC) protocol, KIDSCREEN-10, the Living Healthy Tool (LHT) which was translated and culturally adapted for the Portuguese context, satisfaction questionnaires and focus groups. LHT-student version was administered exclusively in Alter do Chão. Quantitative and qualitative data were analyzed using SPSS v.28.0 and thematic analysis supported by MAXQDA.

**Results:**

Descriptive analyses indicated that 57% of pupils reported irritability or bad mood and 46.7% reported nervousness, while quality of life and sleep were generally favorably rated. Regarding nutritional status, 66% had age-adjusted BMI within the reference range, whereas 36% reported not engaging in regular physical activity. Frequent screen use and exposure to violence during leisure time were also identified as concerns. Community LHT responses indicated differences between municipalities in perceptions of school meals, safety, and access to public services. Focus groups highlighted priorities concerning physical activity, school food environments, and coordination between schools, families, and the community.

**Conclusions:**

The findings indicate context-specific strengths and areas for further attention in child health and wellbeing. Quality of life, sleep patterns, and certain family health practices were generally favorable. Conversely, challenges were identified regarding physical activity, screen time, and psychological symptoms, as well as less favorable assessments of school meal quality, community safety, access to services, and opportunities for active leisure.

## Introduction

Human development is a dynamic, multidimensional, and continuous process shaped by the interaction of biological, cognitive, emotional, and social factors across the life course (1). During childhood, growth and neurofunctional organization support the acquisition of motor and adaptive skills. Higher cognitive functions, including memory, language, and executive processes shape learning and foster progressive autonomy. Emotional experiences and social interactions, in turn, shape behavioral regulation, identity development, and participation in increasingly complex relational environments (1–3). Bronfenbrenner's Bioecological Theory of Human Development provides a useful framework to understand this system. According to the person-process-context-time (PPCT) model, development emerges from reciprocal interactions between the child and the ecological systems surrounding them (4, 5). Proximal processes, understood as regular, meaningful, and sustained interactions between the child and the people, objects, and symbols in their immediate environment, are central drivers of development. These processes unfold across interdependent systems: the microsystem, which includes direct settings such as family, school, and peers; the mesosystem, which refers to the connections between these settings; the exosystem, composed of institutional, community, and organizational structures that indirectly influence the child; and the macrosystem, which encompasses broader sociocultural, economic, and political conditions. The chronosystem further recognizes that individual transitions and historical changes shape developmental trajectories over time (4, 5).

From this perspective, childhood obesity is best understood as a multifactorial phenomenon shaped by the interplay between individual predispositions, environmental opportunities, and structural conditions. Eating habits, physical activity, sleep, screen use, and emotional wellbeing develop within specific contexts, including the family, school, and community (5, 6). These environments influence the opportunities available to children and their adoption of healthy lifestyles. Health behaviors therefore extend beyond individual choices and require analytical models that bring together multiple socioecological factors and the different actors involved in health promotion (5–10). This approach situates the encouragement of children's health within a public health perspective, recognizing that lifestyles established during childhood are associated not only with metabolic outcomes, but also with cognitive and psychosocial health indicators across the life course (11–16).

In Portugal, data from the Childhood Obesity Surveillance Initiative (COSI) 2021–2022 indicate that 31.9% of children aged 7–9 years had excess weight and 13.5% were obese (17). Regional variation was also reported, with corresponding figures of 33.2% and 13.9% in the Alentejo region (17, 18).

Several prevention and health promotion initiatives have been implemented in Portugal, including school-based programs and the MUN-SI intervention model. Findings from these initiatives highlight the potential of multisectoral approaches to foster environments that support child health and wellbeing (18, 19).

Childhood obesity prevention has also been addressed through multilevel, community-engaged initiatives in rural settings. For instance, interventions targeting physical activity and dietary behaviors across individual, organizational, and community levels have demonstrated the potential of ecological strategies while revealing practical implementation challenges, such as facilitator turnover and funding continuity (20, 21). These experiences underscore the value of combining child health assessments with community perspectives and intersectoral collaboration to inform local planning.

To this end, the present study adapted the Grünau Moves good-practice model, a central reference within the HEALTH4EUKids Joint Action, alongside theoretical contributions from Funnell and Rogers, PROMEHS, and Bronstein. Together, these frameworks supported theory-of-change planning, socioemotional skill integration, and intersectoral governance oriented toward shared decision-making and common goals among local partners (8, 10, 22–24).

Grounded in this community-centered approach, this study examined a participatory process of community diagnosis, co-creation, and policy translation for child health promotion across the distinct rural and urban contexts of Portalegre and Alter do Chão. Given the exploratory and observational nature of this work, the study aimed to:

1. Describe biopsychosocial indicators of health, wellbeing, and lifestyles among participating children, considering contextual differences between the rural and urban territories.
2. Examine how children and local actors perceived family, school, and community contexts, identifying resources, vulnerabilities, and opportunities for intervention.
3. Identify priority areas for child health promotion and childhood obesity prevention through triangulation of quantitative indicators, qualitative contributions, and co-creation processes with the community.
4. Reflect on the feasibility and policy relevance of a participatory, multi-informant, and intersectoral approach oriented toward territorial adaptation and potential transferability to other contexts.

## Method

### Study design and setting

This study adopted a cross-sectional observational design within the broader action-research framework of the HEALTH4EUKids project (10), co-funded by the European Commission and focused on child health promotion and the prevention of non-communicable diseases, particularly childhood obesity. Schools were selected through a convenience sampling approach within the established collaboration between the participating municipalities, school clusters, and project partners. In each participating school, all eligible classes in the first cycle of basic education were invited to participate, with participation and refusal rates at the school or municipality level remaining unrecorded.

Implementation took place in the district of Portalegre, covering the municipalities of Alter do Chão and Portalegre, selected because of their contrasting territorial characteristics. The distinction between the municipalities was based on the Urban Areas Typology (TIPAU) established by Statistics Portugal (INE), which integrates population density, land use, and administrative boundaries (25). Within this framework, Portalegre is classified as a moderately urban territory, with approximately 22,000 residents and a population density above 50 inhabitants/km^2^ (25). It centers administrative, commercial, and service functions in the region. Alter do Chão is classified as a predominantly rural territory, with a more dispersed settlement pattern, around 3,000 residents, a low population density below 10 inhabitants/km^2^ with stronger dependence on the primary sector, and predominantly agricultural land use (25).

In addition to the territorial distinction defined by TIPAU, the selection of municipalities also considered local socioeconomic vulnerability. This dimension, assessed through the proportion of children aged 5–11 years receiving School Social Support (ASE), showed relatively high levels in both municipalities compared with the main urban centers in Portugal. This reflects characteristics often associated with inland Alentejo territories, including geographical isolation and lower purchasing power (25).

The regional epidemiological profile was also considered in the selection of the study territories. According to data from the 6th round of the Childhood Obesity Surveillance Initiative (COSI Portugal 2021–2022), Alentejo had prevalences of 33.2% of excess of weight and 13.9% of childhood obesity, slightly above the national estimates (17, 26). Accessibility, operational feasibility, and the existence of established cooperation networks with local and school-based stakeholders were also considered. average.

Given these features, the Grünau Moves model was used as the main reference for structuring the project. The model is guided by community participation, mobilization of local resources, adaptation of interventions to local characteristics and developed to support participatory territorial approaches. Grounded in health equity and sustainability, it supported the definition of actions through co-creation and shared decision-making among partners. Its integration made it possible to adapt the guiding principles of HEALTH4EUKids and the steps of Intervention Mapping to the participating municipalities and to the needs identified locally (10, 22, 27).

### Participatory process, co-creation, and translation into decision-making

A core implementation group was established, comprising the research team and representatives of the main stakeholder organizations, including the participating municipalities and schools, the Portuguese Directorate-General of Health, and the Local Health Unit. The group met monthly throughout the project, both face-to-face and online, and additional meetings were organized whenever implementation decisions or emerging issues required more immediate discussion. The core group supported coordination between sectors, reviewed the progress of implementation, and contributed to the adaptation of the intervention to the local context. In addition, 15 workshops were conducted with teachers, other school staff, and municipal professionals. The workshops differed in their structure and purpose: some were primarily training-oriented, while others followed a more interactive and exploratory format aimed at identifying local needs, discussing priorities, and supporting the co-creation and adaptation of project activities. Differences in stakeholder perspectives were addressed through dialogue and negotiation within the core group, with decisions guided by the project objectives, available evidence, local needs, and implementation feasibility. Although the frequency of the core-group meetings and the total number of workshops were documented, standardized attendance records were not available for every session, which prevents the systematic reporting of individual attendance on a structured table.

The co-creation process followed successive cycles of community diagnosis, discussion, priority setting, implementation, and feedback. Intersectoral meetings, workshops, and sessions with local decision-makers provided opportunities for the joint interpretation of findings and identification of priorities for local action. Scientific coordination ensured methodological consistency, instrument adaptation, data analysis, and the translation of findings into recommendations for local action and policy dialogue.

### Participants

Participants were recruited through convenience sampling, in direct collaboration with the school clusters and local authorities of the municipalities involved. The sample for the assessment of clinical and anthropometric indicators comprised of 202 pupils attending the first cycle of basic education, with a mean age of 7.43 years (SD = 1.14), distributed relatively evenly across the first to fourth grades. Most pupils were male (52.4%) and were all residents and/or students in the municipalities of Portalegre and Alter do Chão.

The number of participants varied across study components according to the scope of each instrument and actual response. The student version of the Living Healthy Tool was administered exclusively in Alter do Chão and was completed by 64 children, with a mean age of 8.30 years (SD = 1.43). Intervention satisfaction was assessed in a subgroup of 61 pupils who voluntarily completed the satisfaction questionnaire. Community version of the Living Healthy Tool was completed by 54 adults from the communities of Portalegre and Alter do Chão, corresponding to the complete responses obtained through online administration of the instrument. This group was predominantly female (96.3%), with a mean age of 48.76 years (SD = 11.51). Regarding occupational status, operational assistants were the largest group (64.5%), followed by teachers (27.8%), health professionals (5.6%), and students (1.8%). Geographically, 57.4% (*n* = 31) reported working or residing in Portalegre and 35.2% (*n* = 19) in Alter do Chão; the remaining participants did not provide this information. The qualitative component adopted an exploratory approach based on four focus groups involving 26 participants, evenly distributed across the two localities (*n* = 13 per locality). Each group comprised 6–7 participants and included representatives from the public and private sectors, civil society, and the wider community in both localities. Participants were purposively selected based on their relevance to the study objectives. Data collection was concluded when successive analyses of the sessions indicated redundancy of the main themes and no emergence of conceptually new contributions relevant to the research questions.

### Instruments

Data collection followed a mixed-method approach, combining quantitative and qualitative components. For the quantitative component, children's biopsychosocial health was assessed using a version of the Health Behavior in School-aged Children (HBSC) protocol adapted for children aged 6–10 years. The instrument covered physical and psychological symptoms, body perception, dietary habits, physical activity, screen use, sleep patterns, interpersonal relationships, and quality of life. Internal consistency of multi-item dimensions was assessed using Cronbach's alpha, with values of 0.77 for psychological symptoms, 0.70 for physical symptoms, 0.72 for problematic screen use, and 0.70 for sleep patterns. For the analysis of activities performed until sweating or becoming short of breath, the responses “once a week” and “never” were grouped as an indicator of low frequency of vigorous physical activity during leisure time. Quality of life was assessed using the KIDSCREEN-10 on a five-point Likert scale. The overall score was used, with higher values indicating more favorable perceived quality of life (28, 29). Nutritional status was assessed using body mass index (BMI), calculated from weight and height measurements obtained through standardized anthropometric procedures. Classification followed the age- and sex-specific international cut-offs of the International Obesity Task Force (IOTF), proposed by Cole et al. (30, 31), categorizing participants as thin, normal weight, overweight, or obese. Within this framework, overweight is distinct from obese, while thinness is defined according to BMI values equivalent to < 18.5 kg/m^2^ in adulthood. Body perception and dieting were assessed using single items from the 2022 HBSC protocol, based on students' reported perceptions and circumstances (28). Health behaviors and perceptions of family, school, and community contexts were assessed using the Living Healthy Tool (LHT), in student and community versions. Developed within the Joint Action Health4EUKids and informed by European good-practice initiatives, including Grünau Moves and Smart Family, the instrument was translated and culturally adapted for the Portuguese context. Responses were recorded on a five-point Likert scale, ranging from “very poor/not at all satisfied” to “excellent/extremely satisfied” (32, 33). The overall LHT score showed an internal consistency of α = 0.76.

Brief satisfaction questionnaires were also administered to students and professionals involved in the activities, addressing perceived acceptability and relevance of the activities and participants' perceptions of their contribution to healthy lifestyle promotion. For the qualitative component, a semi-structured discussion guide was developed for the focus groups. The guide addressed local stakeholders' perceptions of children's behaviors, local health determinants, and factors facilitating or constraining health promotion. It also included questions concerning available territorial resources, community-identified needs, and intervention priorities across family, school, and community settings.

### Data collection procedures

#### Quantitative procedures

Quantitative data collection was carried out in phases. Anthropometric assessment and the administration of HBSC-based indicators took place between November and December 2024, using paper questionnaires.

Weight and height were assessed in person by specialized teams from the Local Health Unit of Alto Alentejo, E.P.E., including nurses and nutritionists trained for these procedures. Assessments were conducted in the school setting using calibrated equipment and standardized anthropometric protocols. Body weight was measured with a calibrated scale, and height was measured using a stadiometer. Children were assessed without shoes and wearing light clothing. The Living Healthy Tool (student version) was administered exclusively in Alter do Chão between May and June 2025 in classroom, with support from parents and caregivers when needed. Although parental or caregiver involvement was not systematically recorded. The community version was made available between December 2024 and March 2025 in digital format through Google Forms. To encourage participation, a QR code was created and disseminated in project materials, municipal social media, communication groups such as WhatsApp, municipalities, health centers, and local commercial establishments. Satisfaction questionnaires were administered in person to pupils and professionals after the activities had been completed. The time interval between the collection of HBSC data and anthropometric measurements and the administration of the LHT should be considered when interpreting the findings jointly.

#### Qualitative procedures

For the qualitative component, participants previously identified for the focus groups were contacted by email and invited to take part in sessions held in reserved rooms provided by the municipalities. Before each session began, the aims of the study were presented, and informed consent was obtained for participation and audio recording. Discussions were facilitated by the principal investigator using a semi-structured guide and lasted approximately 60 min. Audio recordings were subsequently transcribed and anonymized to ensure confidentiality.

### Ethics and data analysis

The study was conducted in accordance with ethical principles applicable for research involving human participants and minors and received prior approval from the Ethics and Deontology Committee (CEDIC; approval no. CEDIC-2025-4-35). Before data collection, written informed consent was obtained from all adult participants and from the parents or legal guardians of participating children, along with their assent. Participation was voluntary, and anonymity and confidentiality were ensured in accordance with the General Data Protection Regulation (GDPR). Quantitative data was processed and analyzed using the Statistical Package for the Social Sciences (IBM SPSS Statistics, version 28.0). Analyses included descriptive statistics, group comparisons using independent-samples *t*-tests and chi-square tests, and exploratory association analyses where appropriate. Parametric tests were used when their assumptions were met. Corresponding non-parametric tests were also performed where appropriate, and when results were concordant, the parametric results were reported. Given the exploratory nature of the project, its cross-sectional design, and the small size of some subsamples, extensive multivariable modeling was not emphasized. Quantitative findings were therefore interpreted primarily as a contribution to community diagnosis rather than as evidence of causal relationships. *A priori* sample size calculation was not performed, and no correction for multiple comparisons was applied. Qualitative data from focus groups and field notes were imported into and organized using MAXQDA 2022. Reflexive thematic analysis was conducted through independent coding by two researchers. Differences in coding were discussed and resolved by consensus. The analysis proceeded iteratively, with data collection considered complete when the main themes had stabilized and no substantively new contributions relevant to the research questions were identified. The interpretation of findings followed a triangulation approach across data sources and methods, considering patterns identified in children's health and wellbeing indicators, community perceptions, focus-group discussions, and satisfaction assessments. This integrated approach enabled the findings to be considered across complementary sources and methods, rather than focusing on isolated statistical associations.

To systematize the methodological information, the different evaluation moments and studies integrated into the project are presented in Table 1, which summarizes participants, instruments, and data collection procedures.

**Table 1:** Methodological characterization of the quantitative and qualitative evaluation of the different studies carried out under the model created in the HEALTH4EUKids project.

Study/evaluation moment	Participants	Instruments	Procedures
Student wellbeing indicators	202 pupils from the first cycle of basic education, from the 1st (27.7%), 2nd (26.2%), 3rd (21.3%), and 4th (24.8%) grades. 52.4% were male. Mean age = 7.43 years (SD = 1.14), between 7 and 9 years. Students in the district of Portalegre (Portalegre and Alter do Chão).	Questionnaire with sociodemographic data, physical and psychological symptoms, quality of life (KIDSCREEN-10), leisure time, problematic screen use, disrespect/teasing, family meals and breakfast, physical activity, body mass index, body perception/diet, eating habits, sleep habits, and chronic illness/disability (8).	Data collected between November and December 2024. Informed consent was obtained from parents/caregivers. Questionnaires were completed on paper with support from parents/caregivers, except for weight and height, which were collected by a team from the Local Health Unit of Alto Alentejo, E.P.E. (nurses, nutritionists, psychologists). Data was analyzed using the Statistical Package for the Social Sciences—SPSS (version 28.0).
Living Healthy Tool—students	64 pupils from the 1st (27.1%), 2nd (22.0%), 3rd (25.4%), and 4th (25.4%) grades. Only pupils from the municipality of Alter do Chão participated in this component.	Healthy Living Questionnaire (Living Healthy Tool), adapted for students, assessing pupils' perceptions in the domains of food, community/school, physical activity/active lifestyle, and sleep. The questionnaire uses a 5-point Likert scale ranging from “very poor/not at all satisfied” to “excellent/extremely satisfactory” (30–34).	Data collected between May and June 2025 in the classroom. Informed consent was obtained from parents/caregivers. Participation was voluntary and anonymous. Data was analyzed using SPSS (version 28.0). Qualitative content was organized in MAXQDA 2022 and examined through reflexive thematic analysis with peer review, ensuring analytical credibility. The process ended after theoretical saturation, with stabilization of themes and no new emerging categories.
Living Healthy Tool—community	54 people who lived or worked in the municipalities of Portalegre and/or Alter do Chão. 96.3% were female. Mean age = 48.76 years (SD = 11.51), between 16 and 65 years. Professionally, 64.5% were operational assistants, 27.8% teachers, 5.6% health professionals, and 1.8% students.	Healthy Living Questionnaire (Living Healthy Tool), assessing participants' perceptions of municipal resources related to the health and well-being of children, young people, and families in the domains of food, community/school, physical activity/active lifestyle, and sleep. The questionnaire uses a 5-point Likert scale ranging from “very poor/not at all satisfied” to “excellent/extremely satisfactory” (30–34). The open-ended response section from the original version was also used to collect suggestions for promoting healthier lifestyles and improving general well-being.	Data collected between December 2024 and March 2025 in the community. The questionnaire was disseminated by municipalities, school clusters, and the Local Health Unit of Alto Alentejo, E.P.E. through school platforms, social media, municipal newsletters, and other local media. Informed consent was obtained from all participants. Participation was voluntary and anonymous. Data was analyzed using SPSS (version 28.0). Qualitative content was organized in MAXQDA 2022 and analyzed through reflexive thematic analysis with peer review.
Student satisfaction	61 pupils from the first cycle of basic education, from the 1st (26.7%), 2nd (23.3%), 3rd (25.0%), and 4th (25.0%) grades. Mean age = 8.30 years (SD = 1.43), between 6 and 12 years. Only pupils from the municipality of Alter do Chão participated in this component.	Questions on satisfaction with school-based activities, assessed on a 3-point Likert scale (dissatisfied, indifferent, satisfied). Pupils evaluated enjoyment of activities, new learning, ease of participation, usefulness for everyday life, enjoyment of activities with peers and adults/technicians, and satisfaction with the spaces where activities took place.	Data collected between May and June 2025 in the classroom. Informed consent was obtained from parents/caregivers. Participation was voluntary and anonymous. Data was analyzed using SPSS (version 28.0). Qualitative content was organized using MAXQDA 2022.
Professional satisfaction	5 professionals, mostly teachers (80.0%). Mean age = 55 years (SD = 13.64).	Satisfaction questionnaire regarding the programmed developed (training and activities). It included items on satisfaction with adequacy, usefulness, and applicability of training content; trainer performance; trainee participation; relationships between participants; and physical spaces.	Data collected between May and June 2025 after program implementation. Informed consent was obtained from participants. Participation was voluntary and anonymous. Data was analyzed using SPSS (version 28.0).

## Results

The quantitative component assessing clinical and anthropometric indicators included 202 pupils from the first cycle of basic education (1st to 4th grade), with a balanced gender distribution and ages ranging from 6 to 10 years. A subgroup of 64 children from Alter do Chão, completed the Living Healthy Tool (student version), and 61 pupils participated in the satisfaction assessment. The community component included 54 participants who lived or worked in the municipalities of Portalegre and Alter do Chão; this group was predominantly female and strongly represented the education sector. The characteristics of the different subsamples are summarized in Table 2.

**Table 2:** Sociodemographic characteristics of the study participants.

Characteristic	Children (Anthropometric sample) (*n* = 202)	LHT subsample (*n* = 64)	Community sample (*n* = 54)
Age (years), mean ± SD	7.43 ± 1.14	8.30 ± 1.43^*^	48.76 ± 11.51
Sex, *n* (%)			
Male	100 (49.5)	–	2 (3.7)
Female	90 (44.6)	–	52 (96.3)
Missing	12 (5.9)	–	–
Municipality, *n* (%)			
Portalegre	106 (52.5)	–	31 (57.4)
Alter do Chão	96 (47.5)	–	19 (35.2)
Not reported	–	–	4 (7.4)
Professional category, *n* (%)			
Operational assistants	–	–	35 (64.5)
Teachers	–	–	15 (27.8)
Healthcare professionals	–	–	3 (5.6)
Students	–	–	1 (1.8)

LHT, living healthy tool; SD, standard deviation.

^*^The satisfaction survey was completed by a subgroup of 61 children from the LHT subsample (mean age = 8.30 ± 1.43 years).

### Student wellbeing indicators

#### Mental health

Most students showed low risk for physical symptoms. However, 35.4% reported tiredness or exhaustion, 14.9% reported headaches, and 10.4% reported stomach aches. In the psychological domain, a moderate overall level of symptoms was observed, especially irritability or bad mood (57%), nervousness (46.7%), fear (39.7%), and sadness (29.8%). Perceived quality of life was generally positive, (*M* = 4.38; SD = 0.49; range = 2.20–5.00). Some less favorable limitations to wellbeing were identified, including difficulties concentrating (27.6%), perceived low school performance (19.5%), lack of personal time (15%), and lack of interesting leisure activities (13.1%).

#### Sleep

Most pupils reported good sleep quality, with an average duration of approximately 10 h per night. Usually bedtime is around 9:30 p.m., and wake-up times around 7:40 a.m. Still, more than 10% reported difficulties waking up in the morning, falling asleep, or having restful sleep. Exploratory group comparisons and correlation analyses did not identify statistically significant associations between screen use and sleep duration or perceived sleep quality.

#### Chronic illness

Approximately 31% of students reported having chronic or long-term health conditions. The most frequently reported conditions were respiratory, food, or medication allergies (11.5%), use of glasses (10.0%), and language difficulties (4.0%). These were followed by asthma (3.5%), psychological health conditions (3.5%), and uncorrected visual difficulties (3.0%). Other reported conditions included hearing difficulties (1.5%); arthritis and motor difficulties (1.0% each); epilepsy, stomach or intestinal disease, kidney disease, and obesity (0.5% each); and other conditions (2.0%). Among students reporting a chronic or long-term health condition, 20% reported difficulties with school attendance, 14% with leisure activities, and 12% with participation in family activities. Students could report more than one condition.

#### Leisure time

The most frequently reported leisure activities, indicated by more than 50% of students, included spending time with family, watching television, listening to music, socializing with friends, sleeping, engaging in sports, and reading. Regarding screen use, 58.4% of students presented indicators consistent with a risk of dependency. During leisure time, 12.0% indicated that they had experienced violence or bullying; perpetration behaviors were not assessed.

#### BMI and eating habits

A total of 66% of pupils had age-adjusted BMI values within the reference range, 19% as underweight, and 15% as overweight or obese. Regarding body image, 75% perceived their body as ideal, 16% as too thin, and 9% as above the desired weight. Approximately 20% considered that they should be on a diet. Concerning eating habits, 33% showed low vegetable consumption, 20% low fruit consumption, and 16% low soup consumption, while 32% reported frequent consumption of soft drinks and 11% excessive consumption of sweets. Most pupils reported eating meals with their family, and 90% reported eating breakfast daily.

#### Physical activity

Regarding physical activity, 49.7% of the students reported engaging in leisure-time activities that made them sweat or become out of breath four or more times per week. In contrast, 36% did not meet the criteria for at least 60 min of physical activity per day during the previous week. Based on the frequency of activities that caused sweating or breathlessness, 22% of the students reported a low-frequency pattern, corresponding to once a week. The most frequently reported activities mentioned by more than 10% of pupils included football/handball, karate, swimming, and dance. The journey to school was mostly by car, with an average journey time of approximately 10 min.

#### Gender comparisons

Comparative analyses revealed statistically significant gender differences (Table 3). Boys showed higher risk of problematic screen use [*t* (177) = 2.57, *p* = 0.006, *d* = 0.51], more disrespect or teasing by peers [χ^2^ (1, *N* = 188) = 5.67, *p* = 0.017], and a higher rate of long-term illness or disability [χ^2^ (1, *N* = 187) = 5.74, *p* = 0.017]. Girls, in turn, reported higher perceived quality of life [*t*(165) = −3.08, *p* = 0.001, *d* = 0.48]. No statistically significant gender differences were found for the remaining study variables.

**Table 3:** Mean comparisons by gender.

Variable	Boys *M* (*n*) or % (*n*)	Girls *M* (*n*) or % (*n*)	*t* or χ^2^/sig.
Physical symptoms	1.22 (94)	1.29 (85)	*t* (177) = −1.01, *p* = 0.077
Psychological symptoms	1.82 (91)	1.68 (84)	*t* (173) = 1.12, *p* = 0.148
Psychophysical symptoms	1.50 (91)	1.46 (83)	*t* (172) = 0.47, *p* = 0.310
Quality of life	4.28 (89)	4.51 (78)	*t* (165) = −3.08, *p* = 0.001^***^
Problematic screen use	1.67 (94)	1.48 (85)	*t* (177) = 2.57, *p* = 0.006^**^
Disrespect/teasing by peers			
No	24.2% (24)	40.4% (36)	χ^2^ (1, *N* = 188) = 5.67, *p* = 0.017^*^
Yes	75.8% (75)	59.6% (53)	
Family meals			
High risk	2.0% (2)	1.1% (1)	*χ^2^* (1, *N* = 189) = 0.25, *p* = 0.617
Low risk	98.0% (97)	98.9% (89)	
Breakfast			
High risk	7.0% (6)	8.6% (7)	χ^2^ (2, *N* = 167) = 1.36, *p* = 0.508
Moderate risk	1.2% (1)	3.7% (3)	
Low risk	91.8% (79)	87.7% (71)	
Physical activity			
High risk	29.1% (28)	26.1% (22)	χ^2^ (2, *N* = 180) = 0.26, *p* = 0.878
Moderate risk	39.6% (38)	42.9% (36)	
Low risk	31.3% (30)	31.0% (26)	
Exercise until sweating or out of breath			
High risk	29.3% (29)	41.5% (37)	χ^2^ (1, *N* = 188) = 0.28, *p* = 0.598
Moderate risk	42.4% (42)	31.5% (28)	
Low risk	28.3% (28)	27.0% (24)	
Body perception			
Thin	16.3% (16)	14.6% (13)	χ^2^ (2, *N* = 187) = 0.11, *p* = 0.945
Ideal	74.5% (73)	76.4% (68)	
Overweight	9.2% (9)	9.0% (8)	
Dieting			
No	79.6% (78)	79.3% (69)	χ^2^ (2, *N* = 185) = 0.49, *p* = 0.783
No, but I should	19.4% (19)	18.4% (16)	
Yes	1.0% (1)	2.3% (2)	
Sleeping well			
Never/almost never	1.1% (1)	2.3% (2)	χ^2^ (2, *N* = 184) = 0.73, *p* = 0.695
Sometimes	8.2% (8)	10.3% (9)	
Almost always/always	90.7% (88)	87.4% (76)	
Long-term illness or disability			
No	63.6% (63)	79.5% (70)	χ^2^ (1, *N* = 187) = 5.74, *p* = 0.017^*^
Yes	36.4% (36)	20.5% (18)	

^*^p < 0.05; ^**^p < 0.01; ^***^p < 0.00.

### Living Healthy Tool—Students

Figure 1 presents the assessment of 16 dimensions related to healthy lifestyle promotion in school, family, and community contexts. The scale adapted for Portugal (0–5 points) identified more favorable and more vulnerable areas within the child health ecosystem. Higher scores reveal indicators aligned with health recommendations, reflecting potentially protective and health-promoting conditions. Intermediate scores identify areas that may benefit from preventive monitoring and improvement. Lower scores indicate less favorable indicators, such as reduced physical activity or excessive behaviors such as prolonged screen use. The LHT–Students results refer exclusively to participants from Alter do Chão.

**Figure 1:**
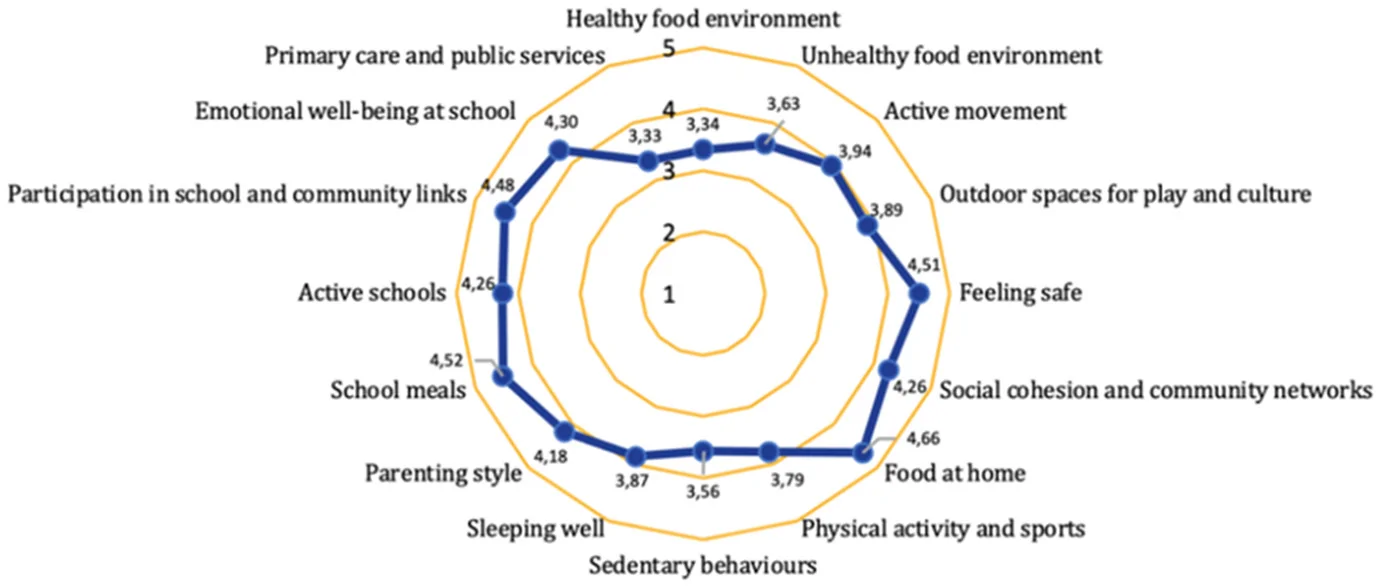
Analysis 1of the 16 dimensions of the Living Healthy Tool—students (Alter do Chão).

The highest scores were observed for food at home (4.66), school meals (4.52), feeling safe (4.51), participation in school and community links (4.48), emotional wellbeing at school (4.30), active schools (4.26), and parenting style (4.18). Intermediate scores were found for active movement (3.94), outdoor spaces for play and culture (3.89), sleeping well (3.87), physical activity and sports (3.79), unhealthy food environment (reverse scored) (3.63), and sedentary behaviors (3.56). The least favorable scores were found for healthy food environment (3.34) and primary care and public services (3.33).

### Living Healthy Tool—Community

Figure 2 presents the assessment of the same 16 dimensions from the perspective of community participants. The highest scores were observed for food at home (4.26), sleeping well (4.18), and parenting style (4.04), highlighting positive contributions from the family context. Intermediate scores were found for feeling safe (3.80), school meals (3.51), participation in school and community links (3.44), and sedentary behaviors (3.49).

**Figure 2:**
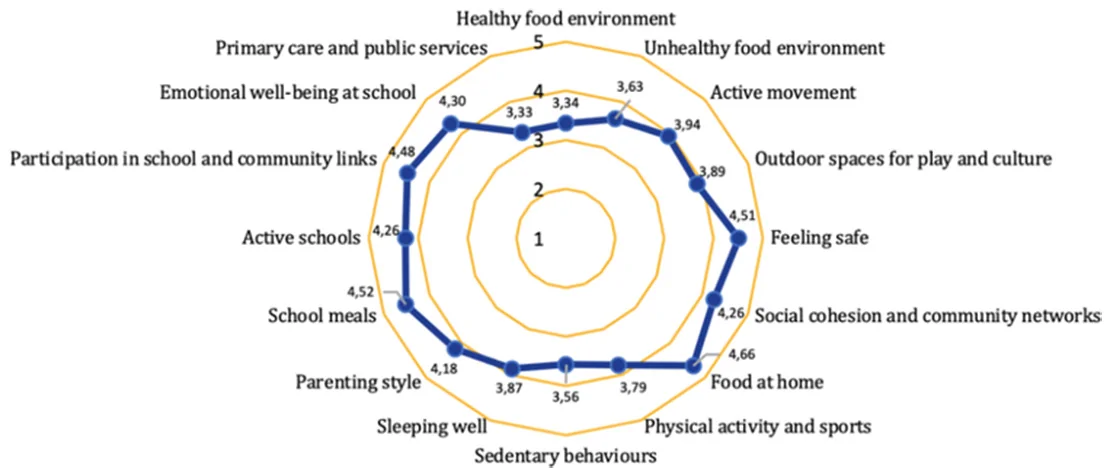
Analysis of the 16 dimensions of the living healthy tool—community.

The least favorable scores were observed for emotional wellbeing at school (3.13), unhealthy food environment (3.17), social cohesion and community networks (3.22), active schools (3.30), and primary care and public services (3.31).

### Comparison between municipalities

Overall, community indicators showed generally positive baseline ratings across the sample (Figure 2). When analyzing by municipality, independent samples *t*-tests revealed statistically significant differences regarding school meals [*t*(51) = −7.79, *p* < 0.001], active school environments [*t*(51) = −2.14, *p* = 0.037], perceived safety [*t*(52) = −2.46, *p* = 0.017], and primary healthcare accessibility [*t*(52) = 2.38, *p* = 0.021], with no other domains reaching statistical significance (Table 4).

**Table 4:** Independent samples *t*-test results for community and school perceptions.

Indicator	Portalegre *M* (SD), *n*	Alter do Chão *M* (SD), *n*	*t* ou χ^2^/*sig*.
Healthy food environment	3.31 (0.83); 35	3.63 (0.90); 19	*t* (52) = −1.30, p = 0.099
Unhealthy food environment	3.03 (1.14); 35	3.42 (0.77); 19	*t* (52) = −1.47, p =0.074
Active movement	3.40 (1.06); 35	3.63 (0.96); 19	*t* (52) = −0.79, *p* = 0.216
Outdoor spaces for play and culture	3.20 (0.90); 35	3.42 (0.90); 19	*t* (52) = −0.86, *p* = 0.197
Feeling safe	3.60 (0.84); 35	4.16 (0.69); 19	*t* (52) = −2.46, p = 0.009^**^
Social cohesion and community networks	3.26 (0.89); 35	3.16 (0.90); 19	*t* (52) = 0.39, *p* = 0.349
Food at home	4.23 (0.67); 31	4.33 (0.62); 15	*t* (44) = – 0.52, *p* = 0.302
Physical activity and sports	3.77 (1.01); 30	3.53 (0.74); 15	*t* (43) = 0.79, *p* = 0.216
Sedentary behaviors	3.61 (0.92); 31	3.21(0.80); 14	*t* (43) = 1.39, *p* = 0.085
Sleeping well	4.19 (0.60); 31	4.14 (0.77); 14	*t* (43) = 0.24, *p* = 0.406
Parenting style	4.13 (0.71); 32	3.86 (0.77); 14	*t* (44) = 1.15, *p* = 0.128
Schol meals	2.85 (0.96); 34	4.68 (0.77); 14	*t* (51) = −7.78, p = 0.001^***^
Active schools	3.12 (0.88); 34	3.63 (0.76); 19	*t* (51) = −2.13, p = 0.019^*^
Participation in school and community links	3.37 (0.73); 35	3.58 (0.90); 19	*t* (52) = −0.92, *p* = 0.182
Emotional wellbeing at school	3.29 (0.93); 35	3.37 (1.12); 19	*t* (52) = – 0.29, *p* = 0.386
Primary care and public services	3.34 (0.87); 35	2.74 (0.93); 19	*t* (52) = 2.38, p = 0.011^*^

^*^p < 0.05; ^**^p < 0.01; ^***^p < 0.001.

#### Community suggestions

The following data come from focus groups conducted in Portalegre and Alter do Chão to explore opportunities and challenges in promoting healthy lifestyles and wellbeing among children and families. Quotations reflect participants' experiences and proposals, with codes assigned by location and group session (e.g., FG_Portalegre_1) to contextualize findings while maintaining participant confidentiality.

“*In schools, attention should be paid to the quality of meals and to supervision in the canteen, as most pupils do not eat soup. There is an urgent need for more human resources to manage conflicts and inappropriate behavior.”* (FG_Portalegre_1)

“*Implement nutrition education programs that involve families. Create and diversify more public spaces for physical activity. Encourage outdoor activities such as walking, cycling, and traditional games to reduce excessive technology use at an early age.”* (FG_Alter do chão_2)

“*Provide more information on bullying, community well-being, autism, and other neurological conditions.”* (FG_Portalegre _2)

“*Promote family walks. Try manual activities, cooking, and making toys.”* (FG_Alter do Chão_1)

“*More school activities during breaks to prevent bullying and negative behaviors.”* (FG_Portalegre_1; FG_Alter do Chão 2)

“*Allow more free time to play, explore the surrounding environment, and strengthen family bonds.”* (FG_Alter do Chão _2)

“*Offer more activities such as swimming, water aerobics, horse riding, and hippotherapy.”* (FG_Alter do Chão_1)

“*Strengthen the involvement of primary care with schools and invest in local infrastructure.”* (FG_Portalegre _1)

“*Promote stronger cooperation between schools, municipalities, and communities.”* (FG_Portalegre _2)

“*There are interesting initiatives underway; however, they often lack enough continuity to produce meaningful and lasting change.”* (FG_Alter do Chão _1)

### Student satisfaction

The satisfaction questionnaire was made available to all students included in the school sample (*N* = 202) and a total of 61 students responded to the questionnaire. Among respondents, 86% reported enjoying the program activities. A total of 92.1% indicated that they had acquired new knowledge related to health and wellbeing, while 88.9% considered their participation accessible. The content was considered useful by 93.5% of s, 98.4% valued collaborative work with peers, 90.5% appreciated interaction with adults, and 98.4% reported satisfaction with the spaces where the activities took place.

### Professional satisfaction

The same questionnaire was also made available to all professionals involved in the program, with five professionals providing responses. Among professionals, 80% considered the training content appropriate, and all participants recognized the usefulness of what they had learned for both their professional and personal contexts. In addition, 80% evaluated the trainers' performance and their own participation in the sessions positively. The group environment was described as collaborative and respectful. By contrast, 60% reported dissatisfaction with the physical facilities.

#### Synthesis of community perceptions and recommendations

An integrated synthesis of findings from different data sources, community perceptions, and intervention priorities is presented across five dimensions: nutrition, physical activity, mental health and wellbeing, community environment, and governance and participation (Table 5).

**Table 5:** Joint display of quantitative and qualitative findings.

Domain	Quantitative and qualitative findings	Integrated interpretation	Implications/identified actions
Nutrition	Family eating routines were favorable, while 15% of pupils had excess weight or obesity. Community concerns focused on school meals, supervision, and vegetable consumption.	Opinions were similar when identifying the school food environment as a priority area, alongside generally favorable family dietary routines.	Higher-quality school meals, nutrition education, and nutrition support through menu planning, meal preparation, and individual consultations.
Physical activity	36% did not meet the 60-min/day recommendation. Participants highlighted outdoor spaces, active mobility, and community activities.	Both strands identified opportunities for physical activity and active mobility as areas for improvement.	Greater availability of structured and informal activity, outdoor recreation, gardens, and routes supporting active mobility
Mental health and wellbeing	Psychological symptoms were reported among pupils. Community participants identified needs related to psychological support, bullying prevention, emotional literacy, and collaboration between schools and families.	Quantitative indicators were complemented by community-identified needs for emotional support and prevention.	Access to psychological support, emotional literacy, bullying prevention, and closer school–family collaboration.
Community environment	Municipal differences were observed in perceived safety, access to services, and conditions for physical activity.	Findings highlighted locally specific environmental priorities rather than a uniform territorial pattern.	Safer, more accessible, and better-maintained public and community spaces.
Governance and participation	Co-creation identified shared priorities involving schools, families, municipalities, and health services.	Participatory processes provided a framework for integrating perspectives from different local stakeholders.	Strengthen intersectoral governance and participatory processes in local health planning and decision-making.

## Discussion

The findings suggest that, despite generally favorable indicators in the family context, particularly around food and sleep routines, important challenges remain in school and community settings. These include limited access to healthy food options, reduced opportunities for physical activity, and weaknesses in social support and health services. Because this was an exploratory, community-based study, the findings are not intended to demonstrate causal intervention effects or identify independent predictors of wellbeing. Their main value lies in the integration of quantitative, qualitative, and participatory evidence to support local diagnosis, priority-setting, and the translation of knowledge into recommendations for municipal policy and action. In relation to food, quantitative and qualitative findings pointed to common concerns about the school context. Focus-group participants stressed the need to strengthen supervision in school canteens and monitor the quality of school meals. Their accounts referred not only to nutritional quality, but also to the presentation of meals, the organization of mealtimes, and adult support for children during meals. Participants also valued appealing to the consumption of soup and vegetables and the educational potential of the canteen as a setting for building healthy eating habits. These observations are consistent with evidence on school food environments, which recognizes the canteen as a context for food learning. The importance of the presentation of meal spaces, repeated exposure to vegetables, and interactions with peers and adults have been associated with greater acceptance of healthy foods and healthier food choices among children (34, 35).

In terms of nutritional status and physical activity, the combined prevalence of excess weight and obesity (15%) and the proportion of pupils who did not meet the recommendation of at least 60 min of daily physical activity (36%) support the relevance of integrated approaches to promoting child wellbeing. These findings underscore the value of considering, beyond individual behaviors, the opportunities and resources available within family, school, and community environments corresponding with European initiatives such as Grünau Moves, Health4EUKids, IDEFICS, and Feel4Diabetes (10, 27, 36–38).

While schools were perceived as supportive settings for physical activity, the quantitative indicators showed that a substantial proportion of pupils did not meet the recommended physical activity criterion. The qualitative findings complemented this information by highlighting the value attributed to outdoor spaces, the use of community resources, and the promotion of active mobility strategies involving schools and families. Although 19% of the sample was underweight, this finding should be considered within the overall distribution of nutritional status and the other health and lifestyle indicators assessed. Available evidence from European countries indicates heterogeneous patterns of thinness across regions and populations. A systematic review and meta-analysis of 49 studies conducted in 26 countries estimated an overall prevalence of approximately 8%−9%, with variation across European regions (39). According to COSI Portugal 2021–2022, the presence of low weight in Alentejo was 1.7% among children aged 6–8 years (17), substantially lower than that observed in the present sample. Such comparisons need to account for age and sex composition, the criteria used to classify nutritional status, and the characteristics of the populations studied. The finding therefore contributes to the characterization of nutritional status in the present sample, while methodological and population differences limit direct comparison with other population estimates.

Regarding socioemotional wellbeing, the quantitative findings indicated the presence of self-reported psychological symptoms, with irritability or bad mood (57%) and nervousness (46.7%) being the most frequently reported. Comparison with national data from the Health Behavior in School-aged Children (HBSC) 2022 study should be considered in relation to the characteristics of the populations represented. The proportion of pupils reporting irritability or bad mood in the present sample (57%) was lower than the nationally reported (71.5%). However, the reference population included pupils in the 6th, 8th, and 10th grades and therefore represented a different age range from that of the present study (28). The observed frequency may reflect characteristics of different developmental stages and the school and social experiences associated with each age group. This comparison contributes to contextualizing the frequency of the indicator; nonetheless, interpretation of the findings in relation to symptom severity and mental health status should account for differences in age and population characteristics (3, 28, 40, 41).

Children and adults in the community differed in their assessment of wellbeing in the school context. This variation may reflect different perspectives on child wellbeing, with children valuing their everyday experiences, peer relationships, and sense of belonging, while adults also considered aspects related to the support available to the educational community, including resources, specialized support, conflict management, mental health promotion, and collaboration between schools and families (42, 43). Considering these perspectives together allows different dimensions of the school environment and educational experience to be integrated, contributing to a broader understanding of child wellbeing (44).

Perceptions of safety in outdoor spaces emerged as another central theme from the qualitative analysis. Participants highlighted concerns regarding public lighting, community policing, and the maintenance of public areas. These qualitative insights align with scores from the community version of the LHT for “feeling safe,” which averaged 3.60 (SD = 0.84) in Portalegre and 4.16 (SD = 0.69) in Alter do Chão (Table 4). This variation reflects heightened concerns in the urban setting compared to the smaller semi-rural context. Reflecting subjective community perceptions rather than official safety records, these insights may contribute to understanding local space utilization. This aligns with evidence linking perceived safety and traffic concerns to children's outdoor space use and independent mobility (45–47).

The comparison between the two municipalities, based on adult community perceptions, provides a descriptive characterization of the patterns identified. Although Alter do Chão received more favorable assessments in some domains, particularly safety, conditions for physical activity, and access to services, this data needs to be interpreted in relation to the specific characteristics of each territory. Studies have shown that child health and development indicators follow different patterns across rural and urban settings, with variation across regions and over time. The global analysis by the NCD Risk Factor Collaboration (48), which included children and adolescents aged 5–19 years across 200 countries and territories, found that the previously observed urban advantage in growth had narrowed in several regions over the three decades analyzed. This pattern was not consistent across populations: while some showed a convergence in growth indicators between areas, others presented more marked contrasts, particularly in parts of sub-Saharan Africa and South Asia (48). Studies from the United States have likewise reported distinct patterns across indicators, with higher probabilities of excess weight or obesity and food insecurity among children and adolescents living in rural areas, but lower probabilities of physical inactivity and high screen use (49–51). Other analyses have identified rural–urban variation in social and emotional protective factors and access to healthcare, indicating that territorial context may be relevant to several dimensions of child health (52). Overall, these findings support consideration of the socioeconomic, environmental, infrastructural, and service-access characteristics of each territory when interpreting the patterns identified, without assuming a uniform direction of health indicators according to geographical location (51–53).

An ecological and participatory perspective on child health promotion and recognizing the complementarity of the contexts in which children live, learn, and develop is supported by the results. Community perspectives also highlighted the role of local resources, infrastructure, and stakeholder involvement in shaping health-promotion priorities. These elements provide a basis for reflecting on approaches that are responsive to the characteristics and resources of different territories.

Results should be interpreted within the context of the study design, sample characteristics, and data-collection procedures. The cross-sectional design does not allow causal relationships to be established between the determinants examined and the observed health profiles. The geographical concentration of the study in Portalegre and Alter do Chão, together with the small size of some subsamples, limits the generalization of the findings to other populations and settings. The student version of the Living Healthy Tool (LHT) was administered exclusively in Alter do Chão, whereas the community version was administered digitally. Participation in the digital community survey may have varied according to access to, familiarity with, and engagement with digital tools. The resulting sample should therefore not be considered representative of the wider community's population. The temporal gap between the collection of anthropometric measurements and the administration of the LHT should be taken into consideration, as this data was not necessarily collected during the same period and should not be interpreted as contemporaneous measures. Parental or caregiver support was permitted when completing some instruments, although the extent of this support was not systematically quantified. This may have influenced responses to sensitive questions, including through social desirability, with possible underreporting of some health-risk behaviors.

Although the frequency of the core-group meetings and the total number of workshops were documented, the absence of standardized session-level records prevented a detailed analysis of attendance patterns, the continuity of stakeholder involvement, and the specific dynamics through which different perspectives were negotiated. The satisfaction findings should be interpreted with caution because participation in the satisfaction survey was voluntary and only 61 of the 202 students in the school sample provided responses, introducing the possibility of self-selection or non-response bias. Similarly, the professional satisfaction results are based on a small number of respondents (*n* = 5), which limits the precision of these findings. These limitations constrain both subgroup comparisons and the generalizability of the results. Additionally, the statistical analysis did not include corrections for multiple testing, which may increase the likelihood of Type I error among the four significant findings reported in Table 3. The findings should therefore be viewed as context-specific evidence that may provide a basis for further research and for the consideration of locally adapted health-promotion strategies.

As a knowledge translation output, a policy brief was developed to summarize the evidence generated by the Health4EUKids pilot in Alter do Chão and Portalegre. Drawing on the biopsychosocial assessment and community mapping, it synthesized the main findings and identified priorities for child health promotion, with recommendations for local and national decision-makers aligned with the National School Health Program (PNSE) and the National Health Plan (PNS).

A policy dialogue was held in Alter do Chão on 14 February 2025, bringing together institutional representatives and community stakeholders from health, education, and local government. The meeting provided an opportunity to discuss the priorities identified through the project and their implications for local planning. Special attention was given to the continuity of intersectoral coordination, evidence-informed planning, and mechanisms for sustained community engagement and monitoring.

The project also generated practical resources, including a health education manual and adapted HBSC items for younger pupils. These outputs may inform future school and community-based initiatives, subject to further validation and adaptation to different organizational and territorial contexts. The experience further illustrates the potential of small-scale, community-based projects as settings for testing and refining health-promotion approaches before considering their application elsewhere.

From an implementation perspective, the experience highlights the relevance of institutional coordination, dedicated human and financial resources, and operational feasibility when considering the continuation or adaptation of such approaches. Any broader implementation should therefore be accompanied by appropriate monitoring and adaptation to the characteristics of the settings involved.

Future studies with larger and more evenly distributed samples across screen-use categories should further examine the relationship between the type, timing and duration of screen exposure and both sleep duration and perceived sleep quality.

## Conclusions

The approach adopted in this pilot study provided an opportunity to explore community-based, co-creative approaches to the identification of child health determinants and the development of locally adapted obesity-prevention strategies. The comparison between municipalities was based on community/adult perceptions, as the student version of the Living Healthy Tool (LHT) was administered only in Alter do Chão. The findings also highlighted the importance of considering territorial characteristics, including community perceptions, infrastructure, and intersectoral dynamics, when defining health-promotion priorities. The project also enabled the adaptation and testing of selected items from the World Health Organization Health Behavior in School-aged Children (HBSC) study for use with younger pupils, supporting their incorporation into the next national HBSC/WHO study (2026–2029). A health education manual was also developed and validated, with potential for integration into the National School Health Program (PNSE).

Broader implementation of this model may present operational challenges, particularly regarding sustained intersectoral coordination and the availability of resources among participating professionals. Any expansion beyond the pilot setting would require further research, continued monitoring, and adaptation to the sociodemographic, infrastructural, and organizational characteristics of different territories. Overall, the Health4EUKids experience illustrates the potential value of collaboration among citizens, professionals, and decision-makers in child health promotion and wellbeing.

## Data Availability

The original contributions presented in the study are included in the article/supplementary material, further inquiries can be directed to the corresponding author.
